# Editorial: Biomarker identification and novel therapies in equine medicine

**DOI:** 10.3389/fvets.2026.1843968

**Published:** 2026-04-28

**Authors:** Agnieszka Żak-Bochenek, Natalia Siwińska

**Affiliations:** 1 Department of Immunology, Pathophysiology and Veterinary Preventive Medicine, Wrocław University of Environmental and Life Sciences, Wrocław, Poland; 2 Department of Internal Medicine and Clinic of Diseases of Horses, Dogs and Cats, Wrocław University of Environmental and Life Sciences, Wrocław, Poland

**Keywords:** biomarkers, colic, horses, osteoarthritis, strangles

Within veterinary medicine, equine research has experienced substantial growth in recent years. This trend is largely due to the evolving perception of horses from primarily being seen as livestock to being valued as companion animals. This shift underscores the need to expand both traditional diagnostic methods and innovative approaches to enhance disease detection, treatment options, and overall equine health management. Despite significant advancements, there remain critical gaps in our understanding of equine diseases, particularly in the integration of traditional and modern diagnostic techniques. Recent studies have highlighted the importance of combining classical diagnostic practices with advanced technologies to improve disease outcomes. However, there is still a need for comprehensive research that bridges these methodologies, particularly in the areas of biomarker identification and novel therapeutic strategies.

Osteoarthritis (OA) is a prevalent and debilitating condition affecting horses across breeds and disciplines. According to a 2013 survey conducted in the United Kingdom, OA was diagnosed in up to 13.9% of the horses examined (1). The proportion of affected animals increases markedly with advancing age, and the disease typically assumes a chronic, progressive course. Numerous studies have therefore focused on elucidating the pathomechanisms underlying intra-articular alterations, with the dual aims of improving diagnostic approaches and developing novel therapeutic strategies (2). The present Research Topic encompasses three articles addressing chronic lameness and OA in horses, underscoring both the clinical importance and the scientific relevance of this condition. In the study by Kwonet al., the effects of an eggshell membrane–based supplement on chronic lameness were investigated. The evaluation employed conventional assessment tools, including subjective gait analysis, rider assessment, joint-angle kinematics, and radiographic examination. The authors demonstrated the feasibility of enhancing these classical methods with advanced counting techniques to standardize and systematize outcome measures. Chabronova et al. presented advanced research on the molecular pathogenesis of equine OA, specifically focusing on the role of small nucleolar RNAs (snoRNAs) and the ribosomal alterations they may induce. Using an experimentally induced model of OA, snoRNAs were identified by high-throughput sequencing. In total, 44 snoRNAs exhibited altered expression across the various comparisons, the majority of which were canonical snoRNAs. These findings indicate that snoRNA expression is dynamically regulated in equine OA, highlighting their potential utility as early molecular biomarkers and as targets for therapeutic intervention. As these studies illustrate, investigation of the pathomechanisms driving joint inflammation frequently relies on experimental models that induce controlled inflammatory responses within the joint. The study by Korac et al. is consistent with contemporary implementation of the 3R (Replacement, Reduction, Refinement) principle in biomedical research, as it aimed to determine a safe and effective concentration of interleukin-1β (IL-1β) for inducing a model inflammatory state.

Among the disorders commonly affecting horses, colic warrants particular attention, as it is most frequently an acute condition characterized by severe abdominal pain and associated with substantial mortality. Colic is estimated to occur in approximately 4–10% of horses annually, most often requiring conservative (medical) management, but in a subset of life-threatening cases necessitating surgical intervention (3), (4). A key objective of contemporary research is the development of rapid diagnostic approaches, especially those capable of guiding the choice of therapeutic strategy. In the study presented in this Research Topic, Crosby et al. evaluated the clinical utility of alactic base excess (ABE) by analyzing data from 538 horses with colic at different stages of disease progression. ABE is derived from blood gas analysis (base excess–extracellular fluid) combined with measurement of L-lactate concentration. While the measurement of L-lactate concentrations in serum and peritoneal fluid is already in clinical use as a biomarker of intestinal ischemia in horses with colic, the authors proposed an extension of this concept (5). Although the investigation did not demonstrate statistically significant differences in ABE values between horses managed conservatively and those requiring surgical intervention, ABE levels were significantly higher in non-surviving horses, suggesting its potential prognostic relevance. Moreover, the study showed significantly elevated L-lactate and glucose concentrations in horses that underwent surgery and in non-survivors, further underscoring the diagnostic and prognostic value of these biochemical parameters.

Strangles, caused by *Streptococcus equi subspecies equi*, is a highly contagious, globally distributed respiratory disease of horses, predominantly affecting young animals (6, 7). The disease is endemic worldwide and is characterized by high morbidity (up to 100% in immunologically naive herds) but generally low mortality, and it frequently causes outbreaks in environments with high animal density (7). Consequently, *S. equi* is a frequent focus of research on rapid diagnostic techniques and preventive strategies, including vaccination (7). Within this Research Topic, we highlight the work of Zu et al., who, for the first time, proposed a method for detecting *S. equi* in specimens such as nasal swabs using an assay that integrates recombinase-aided amplification (RAA) with CRISPR/Cas12a technology. This approach exhibits high analytical sensitivity and specificity. Despite the technological sophistication of the platform, the endpoint readout is straightforward: the presence of *S. equi* can be visually confirmed under ultraviolet light. This feature facilitates potential implementation of the assay in routine veterinary practice.

It should also be emphasized that, as in other animal species, horses are susceptible to neoplastic diseases. Among ovarian neoplasms in mares, granulosa cell tumors (GCTs) are the most frequently reported. The study by Derar et al. outlines current diagnostic and therapeutic options and, through a retrospective analysis, explores potential etiological factors associated with the development of GCTs. The authors describe the role of various imaging modalities and histopathological examination in establishing a definitive diagnosis, and they report a high rate of return to fertility following ovariectomy.

Collectively, the contributions in this Research Topic illustrate the broad spectrum of contemporary equine research, encompassing the application of advanced diagnostic and therapeutic techniques alongside refinement of classical methodologies. The studies discussed herein directly address the central questions posed in the scope of the Research Topic—namely, “How can traditional and modern diagnostic methods be effectively integrated? What are the most promising biomarkers for equine diseases?”—and thereby fulfill its intended aims.
